# Development of transparent eye shields for total skin electron beam radiotherapy

**DOI:** 10.1002/acm2.13722

**Published:** 2022-07-11

**Authors:** J. John Lucido, Andrew J. Veres, Shawn M. Kehret, John A. Angeli, Robert D. Highet, Robert L. Foote, Scott C. Lester, Chris L. Deufel

**Affiliations:** ^1^ Department of Radiation Oncology Mayo Clinic Rochester Minnesota USA; ^2^ Department of Engineering Mayo Clinic Rochester Minnesota USA

**Keywords:** eye‐shielding, patient safety, total skin electron beam radiotherapy, transparent

## Abstract

**Purpose:**

For total skin electron (TSE) beam radiation therapy, the anterior eye and conjunctiva can be protected with eye shields to prevent keratitis, xerophthalmia, and cataractogenesis. Conventional metal eye shields can reduce patient balance by obscuring vision and thus increasing the risk for falls. We report on the design, fabrication, and clinical use of transparent acrylic eye shields for TSE.

**Methods:**

The primary design goals were a seven‐fold reduction in the dose to the anterior eye and conjunctiva to meet published dose‐recommendations, preservation of vision for the wearer, and biocompatibility for external use. Resembling thick swim goggles, the design features 23 mm thick acrylic lenses that are mounted in a 3‐D printed support structure that conforms to the eye socket and can be worn with a strap. Dose measurements were performed in a simulated Stanford‐technique treatment with an anthropomorphic phantom using Gafchromic EBT film

**Results:**

The transparent eye shields were manufactured using a 3D‐printer and CNC‐machine. Based on measurements from the simulated treatments for each of the eye shields, the eye shields provided a 12‐fold reduction in dose to the lens. After use in more than 200 fractions, the shields were well tolerated by patients, and there were no reports of any incidents or adverse events.

**Conclusion:**

Transparent TSE eye shields are able to reduce the dose to the eyes while maintaining vision during treatment at a reasonable cost.

## 1 INTRODUCTION

The use of low‐energy mega‐voltage electron beams to treat a patient's entire skin has a long and successful history in the management of mycosis fungoides, and other cutaneous T‐cell lymphomas.^1^, ^2^, ^3^ Total skin electron (TSE) beam radiotherapy is delivered using large fields at an extended treatment distances using a linear accelerator, often with the electron‐beam's energy degraded below 6 MeV using a low‐Z material.^4^, ^5^, ^6^ While these large fields are effective for treating the patient's skin, the lens, cornea, sclera, and conjunctiva can all be exposed to radiation which can lead to complications, including radiation‐induced cataract, keratopathy, retinopathy, and optic neuropathy.^7^ These regions are often protected by use of eye shields and sparing of the lacrimal glands, eyelids, and eye lashes may be appropriate as well, depending on the extent of involvement of the disease. The European Organization for Research and Treatment of Cancer (EORTC) recommends that the globe receives less than 15% of the dose prescribed to the patient's skin.^1^

Both internal and external eye shields have been used for ocular shielding.^1^, ^5^ Such shields have been made of lead,^4^, ^8^, ^9^ steel,^10^ and tungsten,^9^ which can either be fabricated in the institution or purchased from commercial vendors. Shields made of these materials need only be a few millimeters thick to adequately stop the low‐energy electron beams used for TSE treatments. The low‐profile facilitates their placement behind the skin of the eye lid (i.e., internal shields). While internal shields offer the potential to increase the dose to the patient's eyelids while providing radiation protection to the globes, they are not necessary for patients without evidence of disease in the eyelids and can cause patient irritation and punctate keratopathy.^7^ Additionally, these materials are opaque and block the patient's vision during treatment. This reduces patient comfort and increases the risk of falling during treatment, and may cause some practitioners and patients to opt against using the eye shields. Furthermore, for the commonly used Stanford‐style treatment techniques,^8^ the patients must assume precise poses to minimize the effects of self‐shielding from the extremities to create a uniform dose on the patient's skin.^8^, ^11^ The loss of orientation due to the opaque shields makes it more challenging for patients to maintain positioning which can increase the duration of treatment sessions.

The challenges with opaque ocular shielding for TSE treatments^12^ inspired us to develop transparent eye‐shields that could maintain patient's vision while simultaneously providing adequate radiation protection to the eyes. When necessary, patients receive a boost using orthovoltage therapy—and internal corneal shields—to compensate for the reduced dose to the eyelids and surrounding tissue. We report the design, fabrication, and clinical experience at our institution with transparent eye‐shields for TSE treatments.

## 2 METHODS

### 2.1 Overview of TSE treatment

Patient treatment is delivered using a 6‐field Stanford‐style treatment technique with an extended source to surface distance^1^, ^5^. For each treatment fraction, the patient assumes six poses, each rotated at 60° increments from the previous field. At each pose, two treatment fields are delivered, with the gantry rotated to 20° above and below the horizontal to improve dose homogeneity, totalling 12 treatment fields for each session. The patient was positioned 10 cm behind a polycarbonate scattering panel which is placed approximately 212 cm lateral to the treatment isocenter. A 6 MeV electron beam, which is degraded by the scattering panel to approximately 4 MeV, is used for treatment.

### 2.2 Device design

The primary design goals for the eye shields were a seven‐fold reduction in the dose to the globe of the eye that meets EORTC recommendations, while also maintaining a sufficient field‐of‐view for patient comfort, and biocompatibility for external use. Secondary design goals included low‐cost fabrication, adjustable fit for different patients, ease‐of‐use, and the ability to be cleaned and re‐used.

**FIGURE 1:**
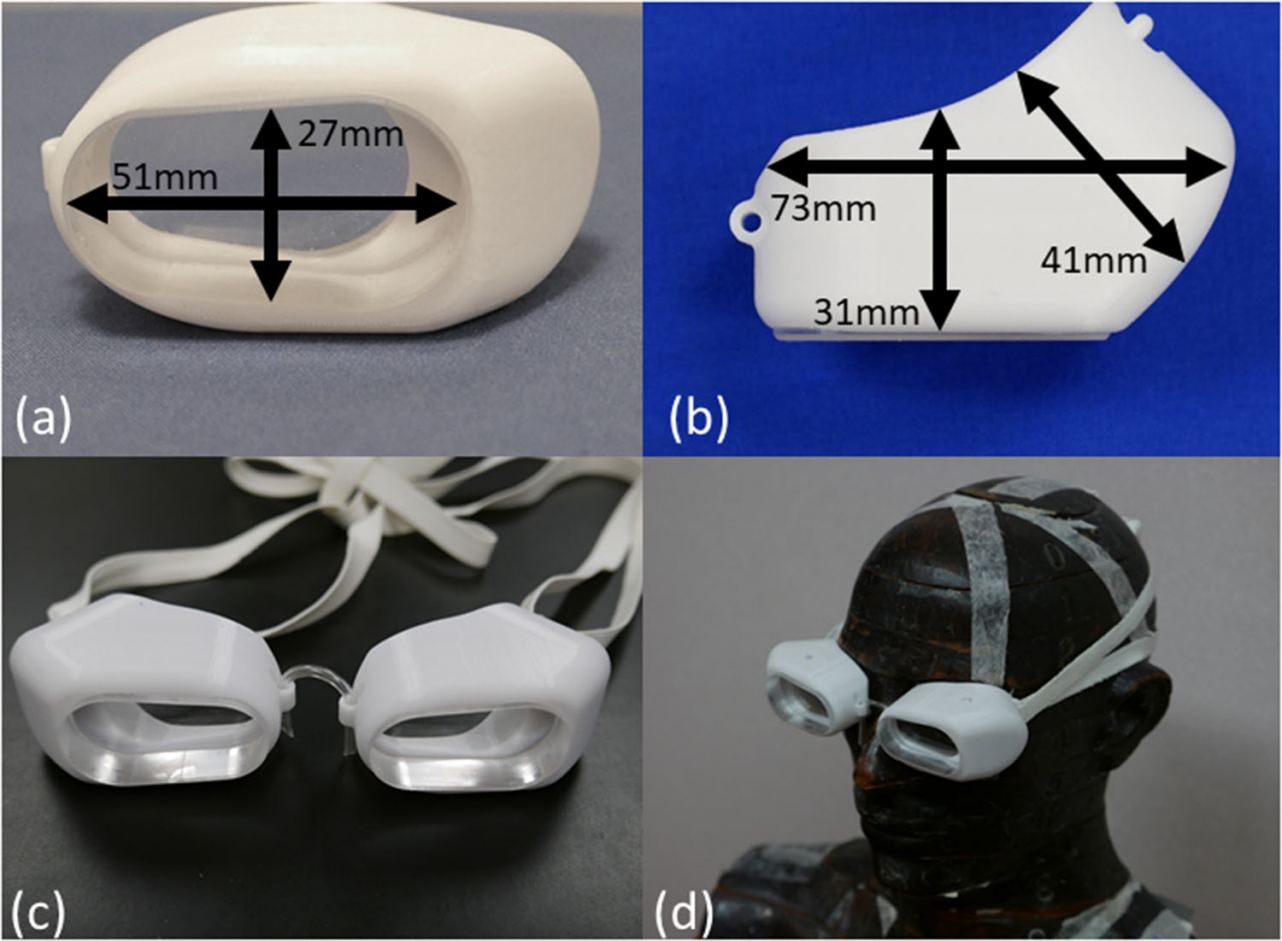
Design and photographs of transparent eye shields for total skin electron (TSE) treatment. Photographs showing (a) front‐ and (b) top‐view of shields marked with measured dimensions, and (c) a fully‐assembled eye‐shield. (d) A photograph of the eye shields placed on an anthropomorphic phantom for in‐phantom testing

The final design of the eye shields is illustrated in Figure 1. The eye shields resemble swim goggles, with two symmetrical eyepieces secured together with a connector over the bridge of the nose, and a thin elastic strap to secure the goggles around the back of the patient's head. Each eyepiece is composed of an acrylic lens mounted in a 3D‐printed support structure. All materials were selected to be of low atomic number to minimize the production of x‐rays from the incident electron beam.

Acrylic was selected for the lens for several reasons. Most importantly, it is transparent and of a sufficiently high density that a reasonable thickness would stop the incident electron beam. In addition, acrylic is easy to machine, widely available, durable and scratch‐resistant if handled properly, and compatible with common cleaning agents. A cross‐sectional thickness of 23 mm for the lens was selected based on the range of the electrons including the beam‐degrading effects of the scattering panel used in our department for TSE treatments.^8^ The lens was machined using a computer‐numerical‐control platform into an approximately oval shape with dimensions selected to give a useful field‐of‐vision for the patient while making sure the profile of the eyepiece does not block surrounding skin unnecessarily.

The 3D‐printed support structure was designed to securely hold the lens in position while providing additional shielding to account for electrons that might travel obliquely from the medial or lateral side of the eyepiece. On the medial aspect of the support structure, an eyelet was placed to enable the nose‐connector to pass through. Another eyelet was placed on the lateral aspect to provide connection to the elastic band to connect to the support structure. We chose to use a 3‐D printable polycarbonate (PC‐ISO, Statasys, Eden Prairie, MN) with full infill. This material had a tensile strength sufficient to withstand the tension used to hold the goggles in place, yet amenable to 3‐D printing with our equipment. These considerations also informed the design of the eyelets. The biggest challenge in the design phase was finding a nose‐bridge piece that was compatible with medial eyelets of a reasonable thickness. It was found that by increasing the thickness of the eyelets and using only silicone tubing (Cole Parmer, Chicago, IL, part# EW‐07407071) over the bridge of the nose better distributed the pressure and protected the eyelet from damage. The nose‐piece was held in place in the eyelet by friction which allows a quick adjustment between the two eyepieces to better fit over the patient's nose, if needed.

### 2.3 Device fabrication

The eye shields were manufactured in‐house. The elastic band used to secure the eye‐shields from the posterior was chosen to be as thin as possible to minimize the perturbation to the beam (Dritz, New York, NY, part# 7704869). This was looped around the patient's head and knotted with appropriate tension once the eye shields were positioned. Photographs of the fabricated eye shields are included in Figure 1.

### 2.4 Quality assurance

An initial assessment of the device was performed prior to first use. The eye shields were visually inspected for defects, damage, and sharp edges that could harm patients or staff, and key dimensions were verified to ensure agreement with the design specifications. Demonstrations and training using the eye shields were performed with treatment staff prior to the first clinical usage, which also served as an initial test of the ease‐of‐use, comfort, and durability.

**FIGURE 2:**
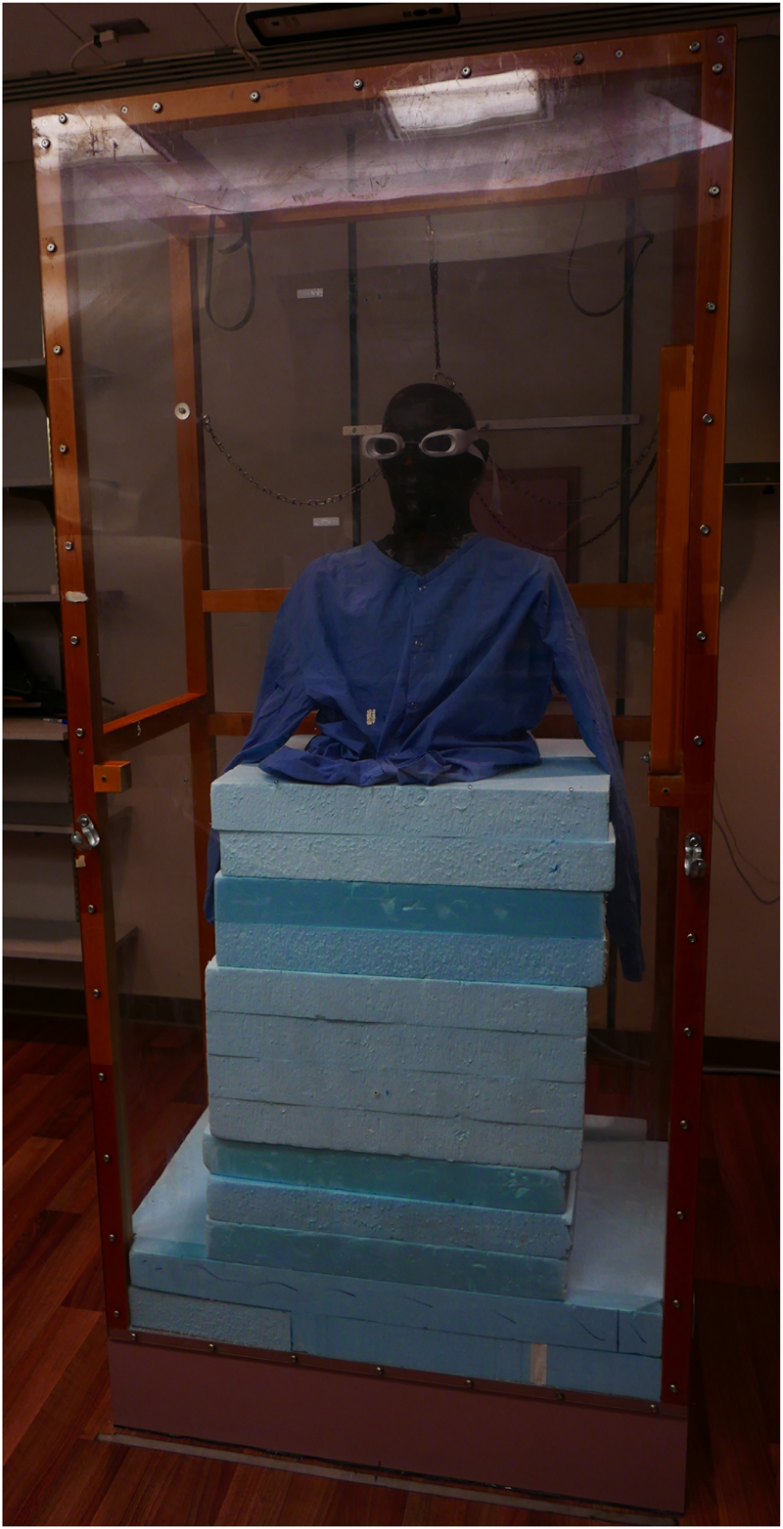
Phantom set‐up for radiation shielding measurements of the total skin electron (TSE) eye shields. Photograph of the set‐up for radiation shielding measurements. The eye shields were mounted on an anthropomorphic head phantom and placed inside the TSE treatment stand with the eye shields are at a height typical for an adult patient

**FIGURE 3:**
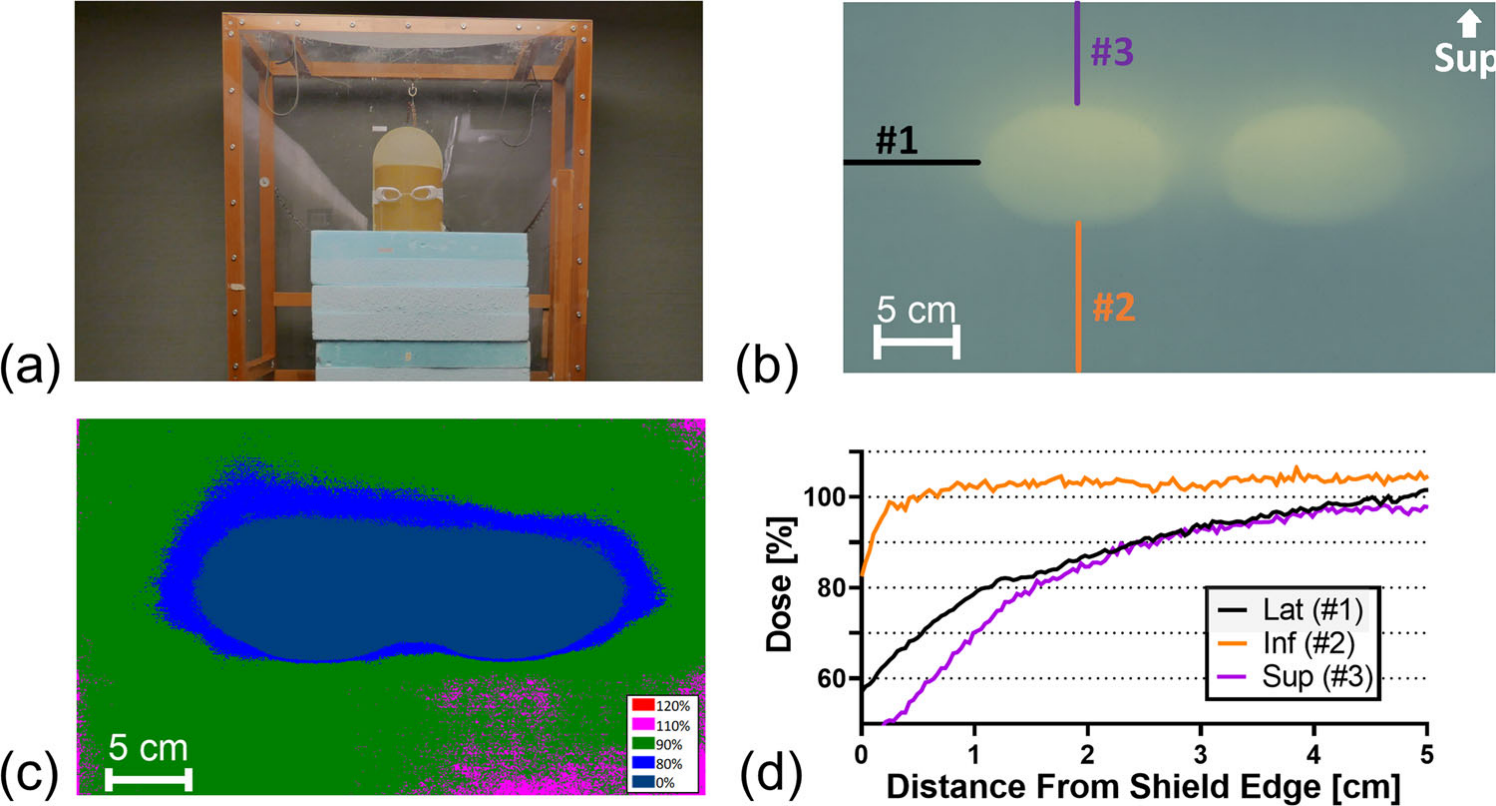
Evaluation of dose reduction in skin surrounding eye‐shields. (a) Photograph of the phantom wrapped in Gafchromic film used to measure the dose distribution around the shields. (b) Image of the exposed film, with lines referring to the location of the profiles obtained: #1, #2, and #3 representing the lateral, inferior, and superior profiles, respectively. (c) 2D dose distribution surrounding eye‐shields, and (d) the dose profiles as a function of distance from physical edge of shield

Radiological testing was performed in order to assess the shielding capabilities of the device. The eye‐shields were mounted on an anthropomorphic phantom as shown in Figure 2, which was then positioned to mimic a patient receiving Stanford‐style treatment. The phantom was rotated through all six Stanford‐style poses, and at each pose the dual‐field beams were delivered. For each pose, a new set of Gafchromic EBT3 films (Ashland, Bridgewater, NJ) was placed on both the inner and outer surfaces of the lens‐pieces to assess shielding of the eye. Additionally, the impact of the eye‐shields on the dose to the surrounding skin was assessed by performing a simulated treatment with the eye‐shields mounted on a cylindrical phantom wrapped in a single sheet of film as shown in Figure 3a.

The absolute dose delivered to the films was measured using FilmQA Pro 2016 software (v5.0, Ashland, Bridgewater, NJ) using a standard film analysis protocol after the film was scanned on an Expression 11000XL scanner (Epson America, Inc., Los Alamitos, CA).^13^ For each eye‐piece, the net attenuation was calculated as ratio of the sum of the shielded doses to the sum of the unshielded doses. Additionally, in vivo measurements were obtained for the first patient who was treated with the eye shields: the Gafchromic films were placed on the inside and the outside of the shields, and the relative transmission was measured for the anterior field.

### 2.5 Ongoing assessment

To assess the durability of the eye shields in clinical practice, the physicists and radiation therapists involved in patient treatments were asked to report any problems with or recommended changes to the eye shields. A hand‐off note was used to record whether any parts needed to be adjusted or replaced, the dates and descriptions of noted issues. The device also visually inspected prior to each use to look for changes that might alter the shielding capability.

## 3 RESULTS AND DISCUSSION

We initially fabricated two pairs of eye‐shields (4 individual eye‐pieces) that passed our initial assessment. The transmitted dose for each eye‐piece was between 5% and 8% for the simulated Stanford treatment delivery (as shown in Table 1), achieving our design goal (and EORTC recommendation^3^) for the dose to the lens. The dose distribution and profiles shown in Figure 3 indicate that the skin surrounding the eye will receive a reduced dose in the area around the shields. Inferior to the device, the dose exceeded 90% of the prescribed dose at approximately 3 mm from the physical edge of the device. Superior and lateral to the device, the dose exceeded 90% of the prescribed dose at distances of approximately 2 cm from the physical edge of the device. This difference in dose based on direction is to be expected: based on the position of the patient's eyes in the field, the beams approach the shields from below, so we expected the beam to be blocked superiorly and laterally to the shields, but not inferiorly. The physician determines whether the eye‐shields are appropriate for a given patient, and whether or not to order a boost to the shielded region. In vivo results from the first patient treatment were consistent with phantom measurements, with a dose measured at 5% of the prescribed dose for the anterior field. It was also observed during patient treatments that the eye‐shields were held securely in place and routine patient motion did not impact shielding or damage devices.

**TABLE 1:** Transmitted dose measurements for eye shields

Eye shield set	Shield	Transmitted dose (%)
Initial	1	5
	2	8
	3	6
	4	7
After cracks discovered	1	6
	2	6
At retirement	1	7
	2	7
Replacement	5	7
	6	7

*Note*: Transmitted dose to the lens with the eye shield in place as a percentage of the dose to the lens without shielding for the simulated patient treatment measured with using Gafchromic film on an anthropomorphic head phantom at treatment position. Four individual eye‐shields were fabricated initially (labeled #1–4), and this table includes the measurements made prior to first use, after the cracks were first found in the support structure for one pair of shields (#1 and #2), as well as after they were taken out of service. The measurements prior to first use of the replacement pair (#5 and #6) are also listed.

**FIGURE 4:**
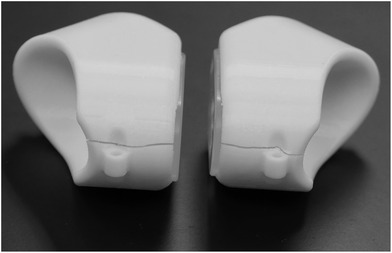
Photograph of the eye shields after cracks developed during routine use. Photograph of the first production units after 6 months (approximately 100 treatments) of use. Cracks had developed in the medial aspect of both units. Film measurements indicated that the cracks did not impact radiation shielding capabilities, but the units were replaced as a precaution against potential structure failure

The device has proven to be durable under routine use. The lenses remain free of any serious scratches or other damage after 18 months of treatment and use in more than 200 treatment fractions. The nose‐bridge piece is replaced after about 25 treatment fractions once it loses elasticity. Additionally, the nose‐bridge piece eyelets have caused discomfort for a small number of patients, which was alleviated by applying a small piece of moleskin to the eyelet. The physicists and therapists noted that hairline cracks developed in the lateral aspects of the support structures after approximately 50 treatment fractions. After approximately 100 subsequent treatment fractions, these cracks had progressively widened, as shown in Figure 4. At this point Gafchromic film measurements were repeated, and demonstrated that adequate shielding was still being provided (Table 1). Nevertheless, replacement eye‐support structures were fabricated because there could be a risk of structural integrity failure at some point in the future. These replacement eye shields were tested prior to use in the same way as the original eye‐shields, and the transmission measurement results were included in Table 1.

## 4 CONCLUSION

This report describes the successful design, manufacture, and clinical use of inexpensive transparent eye shields for TSE radiotherapy. The shields have a swim goggle design with an adjustable fit for different patient shapes and sizes, ease of use, and the ability to be cleaned and reused. The eye shields provide a 12‐fold attenuation of the radiation to the globe of the eye, and their transparency has enabled our practice to routinely provide safe and comfortable eye shielding for all patients.

## CONFLICT OF INTEREST

The authors declare that there is no conflict of interest that could be perceived as prejudicing the impartiality of the research reported.

## AUTHOR CONTRIBUTIONS

John Lucido, Andrew Veres, Robert Foote, Scott Lester, and Chris Deufel were involved in the design, testing, and clinical implementation and use of the devices. John Angeli and Robert Highet were involved in the design and fabrication of the devices. Shawn Kehret was involved in the clinical implementation of the device, providing feedback on the design. All authors were involved in the preparation and review of the manuscript.

## Data Availability

The data that support the findings of this study are available from the corresponding author upon reasonable request.
